# Connecting During COVID-19: A Protocol of a Volunteer-Based Telehealth Program for Supporting Older Adults' Health

**DOI:** 10.3389/fpsyt.2020.598356

**Published:** 2020-12-02

**Authors:** Elena Dikaios, Harmehr Sekhon, Alexandre Allard, Blanca Vacaflor, Allana Goodman, Emmett Dwyer, Paola Lavin-Gonzalez, Artin Mahdanian, Haley Park, Chesley Walsh, Neeti Sasi, Rim Nazar, Johanna Gruber, Chien-Lin Su, Cezara Hanganu, Isabelle Royal, Alessandra Schiavetto, Karin Cinalioglu, Christina Rigas, Cyrille Launay, Olivier Beauchet, Emily McDonald, Dallas Seitz, Sanjeev Kumar, Vasavan Nair, Marc Miresco, Marie-Andrée Bruneau, George Alexopoulos, Karl Looper, Ipsit Vahia, Soham Rej, Syeda Nayab Bukhari

**Affiliations:** ^1^McGill Meditation and Mind-Body Medicine Research Clinic and GeriPARTy Research Group, Jewish General Hospital, Montreal, QC, Canada; ^2^Department of Medicine, Faculty of Medicine, McGill University, Montreal, QC, Canada; ^3^Department of Psychiatry, McGill University, Montreal, QC, Canada; ^4^Institute of Community and Family Psychiatry, Jewish General Hospital, Montreal, QC, Canada; ^5^School of Physical and Occupational Therapy, McGill University, Montreal, QC, Canada; ^6^Douglas Mental Health University Institute, Montreal, QC, Canada; ^7^Department of Epidemiology, Biostatistics, and Occupational Health, McGill University, Montreal, QC, Canada; ^8^Division of Geriatric Medicine, Jewish General Hospital, Montreal, QC, Canada; ^9^McGill University Health Center Research Institute (RI-MUHC), Montreal, QC, Canada; ^10^Division of General Internal Medicine, McGill University, Montreal, QC, Canada; ^11^Department of Psychiatry, Queen's University, Kingston, ON, Canada; ^12^Center for Addiction and Mental Health, Toronto, ON, Canada; ^13^Department of Psychiatry, University of Toronto, Toronto, ON, Canada; ^14^Institut Universitaire de Gériatrie de Montréal, Montreal, QC, Canada; ^15^Department of Psychiatry, Université de Montréal, Montreal, QC, Canada; ^16^Department of Psychiatry, Weill Medical College of Cornell University, New York, NY, United States; ^17^Department of Psychiatry and Harvard Medical School, Boston, MA, United States

**Keywords:** COVID-19, older adults, telehealth, anxiety, depression, geriatrics

## Abstract

**Introduction:** Social-distancing due to COVID-19 has led to social isolation, stress, and mental health issues in older adults, while overwhelming healthcare systems worldwide. Telehealth involving phone calls by trained volunteers is understudied and may be a low-cost, scalable, and valuable preventive tool for mental health. In this context, from patient participatory volunteer initiatives, we have adapted and developed an innovative volunteer-based telehealth intervention program for older adults (TIP-OA).

**Methods and analysis:** To evaluate TIP-OA, we are conducting a mixed-methods longitudinal observational study.

**Participants:** TIP-OA clients are older adults (age ≥ 60) recruited in Montreal, Quebec.

**Intervention:** TIP-OA volunteers make weekly friendly phone calls to seniors to check in, form connections, provide information about COVID-19, and connect clients to community resources as needed.

**Measurements:** Perceived stress, fear surrounding COVID-19, depression, and anxiety will be assessed at baseline, and at 4- and 8-weeks. Semi-structured interviews and focus groups will be conducted to assess the experiences of clients, volunteers, and stakeholders.

**Results:** As of October 15th, 2020, 150 volunteers have been trained to provide TIP-OA to 305 older clients. We will consecutively select 200 clients receiving TIP-OA for quantitative data collection, plus 16 volunteers and 8 clinicians for focus groups, and 15 volunteers, 10 stakeholders, and 25 clients for semi-structured interviews.

**Discussion:** During COVID-19, healthcare professionals' decreased availability and increased needs related to geriatric mental health are expected. If successful and scalable, volunteer-based TIP-OA may help prevent and improve mental health concerns, improve community participation, and decrease healthcare utilization.

**Clinical Trial Registration**: ClinicalTrials.gov NCT04523610; https://clinicaltrials.gov/ct2/show/NCT04523610?term=NCT04523610&draw=2&rank=1

## Introduction

The novel SARS-CoV-2 virus causing COVID-19 emerged in late 2019 (1) and has resulted in 9,660 deaths in Canada and 1.09 million globally as of October 2020 (2). Older adults are overrepresented in the mortality rates, largely due to the higher prevalence of comorbidities and lower immunity seen in this population (3, 4). Globally there are 4.5 billion people in voluntary confinement, including in Canada where the government has urged individuals over the age of 70 to stay at home for the foreseeable future (5). Due to COVID-19 government regulations, community- and residence-dwelling older adults in Montreal are more home-bound; consequently, they may have fewer social interactions or feel more isolated, potentially increasing the risk of stress, depression, anxiety, cognitive decline, and re-hospitalization (6–8). Prior to the pandemic, mental health conditions affected more than 10–15% of older adults (>1,000,000 Canadians) with estimated costs of $15 billion/year (9, 10). The increased vulnerability of the geriatric population during COVID-19 may result in elevated mental health demands, which would further overwhelm the healthcare system (11).

A proposed means of increasing access to mental healthcare during COVID-19 is through telehealth, or the use of technology for remote clinical care and health education (12). Telehealth is a promising avenue for improving symptoms of depression (13) and anxiety (14), and has also been used to connect with socially isolated older adults (12, 15). In one pre-COVID-19 telephoning initiative, older adults formed satisfying relationships, gained confidence, engaged with their community, and became more socially active (15). However, as highlighted by Noone et al., there is a lack of conclusive evidence regarding the effectiveness of video call interventions for reducing loneliness and depression in seniors (16). There is thus a need for more rigorous research with diverse samples to discern whether increased social interaction with volunteers via telephone has the potential to improve psychological outcomes such as symptoms of depression or anxiety.

Currently, global healthcare systems are overburdened, with professionals increasingly becoming burnt out, reassigned, or infected (17, 18). Additionally, most experts agree that a second wave of infection will occur in the fall of 2020 (19), thus implementing community-based volunteer interventions could aid current mental health support services by preventing the development or worsening of mental health issues. For example, in India, a successful mental health intervention with lay volunteers improved depression and other psychiatric symptoms in older adults by providing problem-solving strategies, health education, and assistance in accessing medical and social programs (20). Indeed, not all telehealth modalities may be accessible to older adults (21); one survey found that only 10% used the internet, while 72% owned a cell phone (22). In the context of COVID-19, many telehealth interventions requiring advanced digital literacy are likely unfeasible for rapid implementation due to a lack of infrastructure, training, and low rates of digital literacy in older adults (23). Given these limitations, telephone-based care is likely able to serve the largest proportion of older adults. Thus, we have started a novel volunteer-based telehealth intervention program for older adults (TIP-OA) in Montreal, Canada. The objective of the study is to assess the efficacy of this program by:

1. Evaluating the effects on perceived stress, fear related to COVID-19, symptoms of depression and anxiety in older adults (i.e., clients), as well as the effects on mental healthcare utilization.
2. Comparing clients' mental health outcomes based on baseline risk (i.e., low, medium, or high risk).
3. Analyzing the experiences of clients, clinicians, volunteers, and organizers/stakeholders involved in the program using semi-structured interviews and focus groups.

## Methods and Analysis

### Design

We are offering a large-scale telehealth intervention program, whereby trained volunteers make weekly friendly phone calls to older adults in Montreal, Canada to serve three main purposes:

1. To provide friendly conversation and social connection;
2. To provide information about COVID-19;
3. To connect older adults with services and resources in the community to ensure that the necessities of daily living (e.g., food, shelter, medication, etc.) are met.

The program is a community-based social support intervention that may serve both as prevention and treatment of mental health issues in older adults. In order to evaluate the program and share our findings internationally, we are also conducting a mixed-methods longitudinal study. We are consecutively sampling clients, volunteers, and other implicated parties and collecting both quantitative and qualitative data to understand the impact of this intervention.

#### Telehealth Intervention Program for Older Adults (TIP-OA)

#### Recruitment and Training of Volunteers

Volunteers are recruited (1) using an advertisement circulated on social media; (2) through direct recruitment of medical, social work, psychology, and nursing students from McGill University; (3) from the research and clinical teams' social networks; and (4) through the Jewish General Hospital (JGH) Foundation and Auxiliary, a volunteer non-profit organization. Interested individuals are rigorously screened and interviewed using Zoom^TM^, a widely used video conferencing platform. Selected inclusion criteria are the ability to (1) commit for a minimum of 3-months; (2) provide this service to 3-5 clients; and (3) call each client at least once every week. Applicants with a criminal record or applicants who refuse to submit to a background check are excluded from volunteering.

Members of the volunteer management team stratify volunteers according to past volunteering and clinical experience(s) (Appendix A in Supplementary Material). Two trainers facilitate two weekly 2-h training sessions over Zoom^TM^, to a maximum of five volunteers per session (please contact info@whatconnectsus-cequinouslie.org for more information regarding the training design and/or manual). The initial virtual training was provided in kind collaboration with the Touch-Volunteers in Partnership (ViP) program at the Jewish General Hospital. The Touch ViP program was created through a participatory mixed-methods study, which engaged persons living with mental health conditions, their families, and mental health professionals over 3 years to support recovery-oriented practices (24, 25). The Touch Volunteer Manual, originally designed for in-patient services, was adapted for telehealth and is provided to TIP-OA through the *What connects us-Ce qui nous lie project* Dementia Community Investment fund (1920-HQ-000092), to support the aim to create a web of inter-sectoral resources and decrease stigma. In addition to the stratified matching, the program has been further modified for TIP-OA to address the needs of older adults during COVID-19 with feedback from trainers, clinicians, and volunteers.

The volunteer training covers topics such as client consent, confidentiality, active listening, responding to possible suicide risk, community resources (e.g., grocery delivery and hotline services), and common scenarios that may come up during calls. The training also includes basic information about psychotic features and instructs volunteers to reach out to the clinical team if they require extra support in interacting with their client, or if they have concern for the safety and well-being of their client or someone else. The design of this training informs the follow-up methodology used to support volunteers, with an emphasis on stigma reduction as a means toward interpersonal connection. Similar to Touch, the training and implementation are iterative and responsive to the needs of volunteers and clients.

#### Client Referrals, Screening and Matching

Clients are either self-referred or referred from the JGH (COVID-19 inpatient units or the geriatric psychiatry clinic), long-term care facilities, local primary care clinics, or community organizations in the west-central Montreal healthcare/administrative catchment region (which represents over 500,000 residents from diverse ethnic backgrounds). Interested clients and clinicians can call our voicemail number, send an email, or fill out a form on our website to refer to our program. Included clients are consenting older adults (age ≥ 60) living in Montreal. Excluded individuals (1) are actively suicidal, (2) have a severe hearing impairment or other disability preventing the use of a phone, or (3) are suffering from moderate or severe dementia, as determined by clinical judgment (e.g., based on client history and/or past memory testing), or (4) are unable or unwilling to provide informed consent.

At intake, the screening clinician or trainee (e.g., in psychiatry, social work, psychology) spends 20–30 min on the phone with each client to collect basic demographic information and perform an informal clinical assessment regarding the client's level of cognitive functioning (coded as having suspected mild dementia or not). In cases where the client was referred from a clinic, our clinical team receives information from the referring clinician regarding the clients' cognitive and overall functioning. The screening clinician also assesses the client's level of risk and the complexity of their case, conducting a brief assessment developed primarily from Culo's paper (26) and national guidelines pertaining to seniors' mental health (27). This risk assessment is flexible and allows for the consideration of factors that are not included in the assessment, but that may hamper communication between the client and volunteer. The team then stratifies clients into categories based on case complexity and the initial risk assessment (Appendix B in Supplementary Material). Although we only assess case complexity at baseline, communication with volunteers through various channels (see section Volunteer Support and Quality Assurance) typically informs us of client mobility between categories. Following client screening and categorization, a clinician and volunteer manager assign clients to newly trained volunteers according to their assigned categories on a weekly basis.

#### Volunteer Support and Quality Assurance

Multiple systems are in place for volunteer support and quality assurance:

- *Follow-up meetings:* The training team hosts weekly drop-in follow-up meetings for volunteers on Zoom^TM^. Trainers answer volunteers' inquiries and facilitate learning through group discussion and problem solving. Training and implementation can also be responsive to the emergent concerns and situations from these follow-up sessions. Although we encourage all volunteers to attend a follow-up meeting with the trainers, we emphasize to code red volunteers that they should refer to these sessions for extra support in managing their clients' cases.
- *Volunteer hotline:* Volunteers are provided with a phone number to call during working hours (9 a.m.−5 p.m., Monday through Friday) should they have clinical and/or urgent concerns regarding a client. An automatic system connects the volunteer with the clinician on-call for rapid assistance. The clinician support services are provided by professionals and advanced trainees with considerable experience in geriatric mental health (e.g., in the fields of geriatric psychiatry, clinical psychology and neuropsychology, occupational therapy, social work, and nursing).
- *Volunteer email address:* Volunteers are given an email address to contact in the case that (1) their client does not wish to receive the service anymore, (2) the volunteer would like to take on another client, or (3) the client/volunteer would like to be paired with someone new. A research assistant checks this email daily.
- *Client voicemail:* Upon entry into the telehealth intervention, a clinician provides clients with a voicemail number to contact should they have any queries (e.g., issues with their volunteer or the delivery of the service). The trainers instruct volunteers to additionally give this number to clients during their first contact. A team member checks this voicemail on a daily basis and relays the messages to the appropriate person. This voicemail is also used for clients who self-refer to receive TIP-OA.

### Interventional Methods

TIP-OA involves 1–2 times/week friendly phone calls from trained volunteers to adults >60 years old. Although the calls serve primarily to provide friendly conversation, they also grant volunteers the opportunity to inquire about clients' general well-being, provide information about COVID-19, and assess basic needs the client may have. If needed, volunteers refer clients to community resources and services (e.g., grocery or medication delivery, hotlines). In this way, the friendly phone calls may prevent against the worsening or development of mental health issues or may treat pre-existing or recent mental health conditions.

### Selection/Treatment of Subjects

The first 200 TIP-OA clients who provide informed consent will participate in data collection for research.

#### Exposure

The client exposure for the quantitative portion of this study is the TIP-OA intervention.

#### Client Quantitative Outcome Measures

All primary and secondary outcomes will be assessed over the phone at baseline, 4-, and 8-weeks (primary study endpoint):

##### Primary outcome

*The Perceived Stress Scale (PSS)* is a validated and commonly used measure of stress (28). The 14-item scale assesses the degree to which life events are appraised as stressful. It asks respondents about how often they have felt certain ways in the past month, with responses ranging from 0 (never) to 4 (very often).

##### Secondary outcomes

*The COVID Fear Scale* is an 18-item scale measuring individuals' anxiety, fear, and concern surrounding the COVID-19 pandemic. Items include: “Fear that I will be infected” and “Worry if I will be assigned to COVID wards if hospitalized” (29). The *Patient Health Questionnaire-9 (PHQ-9)* is a 9-item self-report questionnaire used to assess depressive symptom severity. The questionnaire asks individuals how often in the last 2 weeks they have been bothered by problems like “feeling down, depressed, and hopeless” and “poor appetite or overeating.” Scores for each question include 0 (not at all), 1 (several days), 2 (more than half of the days), and 3 (nearly every day) (30). The *Generalized Anxiety Disorder 7-Item Scale (GAD-7)* is a brief scale that measures symptoms of anxiety present in the previous 2 weeks. Respondents' scores can range from 0 (not at all sure) to 3 (nearly every day). Items include “Not being able to stop or control worrying” and “Being so restless that it's hard to sit still” (31). *Mental healthcare utilization* will be assessed using medical records, including the number of ER visits, hospitalizations, and outpatient visits (1) in the 8 weeks prior to baseline and (2) during 8-week follow-up.

##### Additional measures/covariates

*Baseline risk:* during the screening process, our clinician team codes clients as green (low risk), orange (medium risk), or red (high risk) (Appendix B in Supplementary Material), chiefly in order to match clients with appropriately skilled and experienced volunteers. Demographics: Age, living situation, education, mental illness diagnoses, and psychotropic medication use will be obtained at baseline. *Digital Literacy:* We will systematically measure digital literacy using the Technology in Geriatric Psychiatry scale, which is a 14-item questionnaire about patients' access to technological devices, comfort or familiarity with software, availability of family members who can assist in setting up technology, interest in learning to use video conferencing software, and interest in receiving support via videoconferencing. This scale was developed by co-investigator Dr. Ipsit Vahia from Harvard University and is currently being validated in another study.

#### Client, Volunteer, and Stakeholder Qualitative Outcome Measures

*Zoom*^*TM*^ *focus groups* will be conducted at an 8-week follow-up with 16 volunteers (eight per group) and eight clinicians/stakeholders, who will be consecutively selected and invited over the phone to participate. Verbal informed consent will be obtained before the start of the focus groups.

*Semi-structured interviews:* 10 different stakeholders (e.g., referring clinicians, clinical team, coordinators, community partners), 15 volunteers, and 25 clients (consecutively selected) will additionally be invited to participate in individual semi-structured interviews, for which verbal informed consent will be obtained. The client interviews will consist of open-ended and guided questions regarding the overall program and its impact on their mental health and well-being (see Appendix C in Supplementary Material). The main purposes of the volunteer and stakeholder focus groups and interviews are to analyze their experiences (including challenges and opportunities encountered) and to assess the strengths, weaknesses, and viability of the program. For example, volunteer focus groups and interviews inquire about the types of issues encountered when dealing with clients, their ability to provide clients with support, and their experience accessing support from the TIP-OA team. Stakeholder focus groups and interviews include questions about their comprehension of the program procedures, their perception of the program's impact on clients, and their ability to work with the TIP-OA team.

### Data Analysis

#### Quantitative Analysis

Descriptive statistics for continuous variables will include means and standard deviations, while categorical variables will be summarized using frequencies and proportions. A within-subjects repeated measure analysis of variance (ANOVA) will be conducted for the PSS (primary outcome), COVID Fear Scale, PHQ-9, GAD-7, and mental healthcare utilization (secondary outcomes) to assess differences in measures between baseline and the 8-week follow-up between groups with different baseline risk (low, medium, and high risk). Additional analysis of covariance (ANCOVAs) will control for variables that differ between groups at baseline. We will use LOCF to handle missing data that results from study attrition. Although we have found it extremely rare to be missing baseline data when, for example, a participant answers all but one question on a measure, in any such case we will exclude the participant's data for that measure. All ANOVAS will be tested at two-tailed alpha = 0.05. Linear mixed effect regression models, adjusting for age and sex, will analyze the effect of TIP-OA on all outcomes (PSS, COVID Fear Scale, PHQ-9, GAD-7, and mental healthcare utilization) collected at baseline and 8-week follow-up. We will perform additional linear mixed effect regression models accounting for a larger group of important variables such as client referral source (e.g., referred from clinic vs. self-referred) and cognitive functioning (e.g., suspected mild dementia vs. no suspected mild dementia) on mental health outcomes. Variable selection in linear mixed effect model (32) will be used to select important fixed effect covariates as they relate to the outcome. The fixed effect point estimates and corresponding confidence intervals and *p*-values will be computed. Additional subgroup analyses will also compare the primary outcome (PSS) between important subgroups [e.g., referral source (referred from clinic vs. self-referred) and cognitive functioning (suspected mild dementia vs. no suspected mild dementia)]. Analyses will be run using R statistical software.

#### Qualitative Analysis

A descriptive phenomenological approach will be used to analyze qualitative data. This descriptive thematic analysis will focus on the lived experiences of clients, volunteers, and stakeholders in order to map out the broader phenomena emerging from TIP-OA. We will code data based on repeated patterns of words, key-words-in-context (KWIC), contrasting information, and by searching for missing data. These patterns will help to identify and organize themes based on frequency, recurrence, and complexity of meanings in the data. Using a gender lens, participant narratives will be analyzed to understand their experiences. Coding will take place in three phases. Phase 1 will be descriptive, classifying the participants' narratives into standardized, predefined codes. In phase 2, descriptive coding will inform categorical coding by helping to further define data categories. In phase 3, analytical coding will allow interpretation of data-grounded emergent themes. To establish and authenticate links between different sources, we will conduct data triangulation by approaching individual narratives from various sources. We will use NVIVO (33) to establish frequencies of codes within narrations along with the source, quality, and depth of the content.

### Sample Size Justification

Comparable intervention studies for late-life stress, fear, depression, and anxiety have detected statistical significances and clinically meaningful effects (20, 34) including one volunteer-based program with a sample size of 150–200, which showed efficacy in preventing episodes of major depression in older persons with subsyndromal symptoms (19).

## Results—Early Experience

As of October 15th, 2020, 150 volunteers have been trained to provide TIP-OA to 305 older clients. Final research ethics approval has been provided and we will consecutively recruit 200 clients for quantitative data collection. After 8-weeks, 16 volunteers and eight clinicians will be consecutively selected for Zoom^TM^ focus groups, and 25 clients, 15 volunteers, and 10 stakeholders will be selected in the same manner for semi-structured interviews on Zoom^TM^.

## Discussion

Through regular friendly phone calls and the development of relationships between older adults and trained volunteers during the COVID-19 pandemic, TIP-OA aims to not only improve geriatric mental health symptoms, but also prevent mental illness in a wide sample of older adults. Crucially, many of the design elements (collaboration of volunteers, iterative design and implementation of training and follow-up, ease of referral) are geared toward long-term sustainability. Our longitudinal mixed-methods evaluation of TIP-OA's effectiveness, viability, and impact will inform future systematic telephone-based support for older adults by identifying roadblocks, thus allowing for rapid mobilization of quality telehealth services in response to future crises. Depression and anxiety symptoms in adults during COVID-19 in China have been estimated to be 15.0 and 30.9%, respectively (35), compared to pre-pandemic estimates of 3.6 and 5.0% (36). Notably, during the SARS epidemic in Hong Kong, geriatric suicides increased by 31.7% (37) likely due to low mood and stress related to social isolation (38). During SARS, while healthcare service utilization in most domains decreased, utilization of mental health services did not (39). These studies emphasize the urgent need for low-cost, scalable, and convenient interventions to mitigate the deleterious effects of fear, anxiety, and depression on the already vulnerable geriatric population. Research suggests that interventions like TIP-OA may alleviate the burden on healthcare infrastructure by providing a method for managing and preventing mental health issues which could decrease the number of clinical mental health services requested (20).

Older adults, especially those with pre-existing mental health conditions, have so far fared relatively poorly during COVID-19 (40), but pre-COVID-19 telephoning initiatives indicate that regular telehealth communication may facilitate connection to others and their community and enhance confidence (15), factors known to protect against mortality and morbidity (41). A recent study showed that the 12-month incidence of major depression (4.9%) in subsyndromal older adults was significantly less in those receiving regular lay-person phone calls compared to treatment as usual (12.6%) (20). The mean 12-month incidence of depressive symptoms was also significantly lower (−1.18 on the 12-item General Health Questionnaire) (20).

TIP-OA has a similar structure to previous telehealth interventions but may, in the context of the COVID-19 pandemic, show a greater positive impact on mental health due to the government enforced social distancing. Compared to similar studies, our mixed-method data collection and our broad variety of mental health outcomes (stress, depression, anxiety, mental healthcare utilization), will give a more comprehensive picture of TIPOA's impact on clients' mental health. Despite taking years to implement, typically therapeutic mental health clinical trials produce evidence for narrow populations (often circumscribed to a single mental health diagnosis) due to extensive exclusion criteria that may disqualify up to 85% of people in depression trials (42) and up to 92% in anxiety trials (43). In light of the pandemic, TIP-OA may fulfill the need for large-scale public mental health interventions that address both the prevention and treatment of mental health conditions in a wide and diverse sample, with the goal of rapidly and efficiently generating real-world generalizable evidence for dissemination. Furthermore, TIP-OA's focus on the development of relationships through friendly conversation and the assurance of necessities may yield greater positive impacts on mental health variables. Lastly, the streamlining of volunteer tasks will offer more time to address daily life- and mental health-related concerns as they arise.

## Strengths and Limitations

TIP-OA can be a large-scale low-cost telehealth intervention that leverages lay-volunteers to support geriatric mental health. A foundational component of TIP-OA, the volunteer training, resulted from long-term participatory research with patients and other stakeholders in psychiatry, and thus promotes important aspects of mental health recovery including Connectedness and Empowerment, derived from the CHIME model (44). Furthermore, the use of peer support fosters positive social relations between volunteer and client, enhances the development of social networks, and empowers the client in maintaining and/or improving their mental health (45, 46). Volunteer training that curtails clinical intervention and facilitates the development of non-hierarchical relationships may enhance the connection between volunteer and client and mitigate certain aspects of client decompensation through personal empowerment. Lastly, our large heterogeneous sample will maximize generalizability, informing the development of a model for scalable public mental health prevention and treatment that accommodates seniors with different backgrounds and mental health concerns.

The main limitation is that this study is non-blinded, non-randomized, and observational. If funding permits, we plan to conduct a pilot randomized controlled trial comparing outcomes of TIP-OA vs. a waitlist control. Another limitation is that our system for categorizing clients (Appendix B in Supplementary Material) is imperfect; although we based it off of previous literature and guidelines on geriatric mental health (26, 27) and it serves our purposes of facilitating appropriate matching between clients and volunteers, it does not account for the reality that people present with different types of risk. What's more, different risks may not map perfectly onto the categories that we developed. We were also not able to account for changes in risk category over the follow-up given that we only assessed client risk at baseline. It is possible that over the course of the 8 weeks, some clients moved between risk categories. Future research may consider using a universal, standardized risk assessment measure for coding clients' risk/case complexity at baseline and at the follow-up. Another potential weakness of this study is the lack of a structured cognitive assessment. We did not include a standardized measure as it would be challenging to administer over the phone with our limited human resources and time constraints. That said, our approach of documenting instances of clinician-suspected cognitive impairment is constructive in light of the aforementioned challenges, especially considering that several studies in the field of telehealth for geriatric mental health have not documented cognitive status in their reports (15, 47, 48).

## Ethics and Dissemination

Expanding TIP-OA into populations with diverse needs and cultural sensitivities requires relevant understanding within the volunteer pool. Thus, our expansion efforts will focus on offering and adapting the program to community organizational frameworks so that TIP-OA acknowledges and integrates the structures, experiences, and knowledge embedded within communities. If our project is successful, we will disseminate knowledge about TIP-OA by conducting workshops and establishing partnerships, with the ambition of implementing it across Canada and internationally. Future directions will also include cost-effectiveness evaluations of similar interventions in other vulnerable populations, the development of a randomized controlled trial design, and the broadening of telehealth resources such as webinars and remote group therapy. Future research may benefit from examining the effect of similar telehealth initiatives on other psychological factors such as quality of life, social connectedness, and resilience.

## Concluding Remarks

This program represents one of the largest and most systematic, evidence-based initiatives to address the mental health of older adults in the province of Quebec during the COVID-19 pandemic. It has the potential to significantly improve mental health outcomes for hundreds of clients and will serve to define the rapidly increasing prominence of telehealth in the future of mental health prevention and care far beyond the current global crisis. In light of this, it will be crucial to identify and specify the most effective interventions, both through evaluations of cost and the systematic, randomized and blinded measurement of outcomes.

## Ethics Statement

The study involving human participants was reviewed and approved by JGH Research Ethics Committee. Verbal informed consent (recorded) for participation was required for this study in accordance with the national legislation and the institutional requirements. Recorded verbal informed consent to participate in this study will be obtained from the patients/participants, and a copy of the consent form will be mailed or emailed to each participant according to their preference.

## Author Contributions

ElD contributed to the design of the volunteer management protocols, to the direction of the volunteer management team, and to the conception and writing of this manuscript. HS led the research development of this project and played a major role in the writing of this manuscript. AA contributed to the clinical components of this project and to the editing of this manuscript. BV, AG, EmD, IR, and AS developed the clinical protocols for this program and provided feedback on this manuscript. PL-G contributed to the clinical duties of this program and to the revision and submission of this manuscript. HP, RN, JG, KC, and CR contributed to the conception and implementation of the volunteer management, community resource, and research components of TIP-OA. CW developed the volunteer training component and along with NS, JG, HS, KC, and PL-G trained the TIP-OA volunteers. AM, CH, CL, OB, EM, DS, SK, VN, MM, M-AB, GA, KL, and IV all provided important revisions on this manuscript. C-LS determined and verified the analytic methods. SR and SB were responsible for the overall vision, conception, implementation, and supervision of the presented idea. All authors contributed to the article and approved the submitted version.

## Conflict of Interest

DS is a site investigator on a clinical trial sponsored by Hoffmann La Roche. VN receives research funding from Brain Canada, Eli Lilly, as well as Merck & Biogen Pharmaceutical companies (project completed). He declares no influence or impact on the project under consideration. SK receives research support from Brain and Behavior Foundation, National Institute on Aging, BrightFocus Foundation, Brain Canada, Canadian Institute of Health Research, Center for Aging and Brain Health Innovation, Center for Addiction and Mental Health, University of Toronto. He also receives equipment support from Soterix Medical. GA served on the Advisory Board of Eisai and of Janssen Pharmaceuticals. He also served on the Speakers Bureaus of Allergan, Otsuka, and Takeda-Lundbeck and his work was supported by P50 MH113838. SR receives a junior investigator salary award from the Fonds de Recherche Quebec–Santé and an investigator-initiated research grant from Satellite Healthcare (Dialysis Company) for an unrelated project. The remaining authors declare that the research was conducted in the absence of any commercial or financial relationships that could be construed as a potential conflict of interest.
