# Supplementary material for: Connecting During COVID-19: A Protocol of a Volunteer-Based Telehealth Program for Supporting Older Adults' Health
Source: Front Psychiatry. 2020 Dec 2;11:598356. doi: 10.3389/fpsyt.2020.598356 (PMC7738321; doi:10.3389/fpsyt.2020.598356)
Supplement: Supplementary file 1 [file Data_Sheet_1.PDF]

**Appendix A. Categorization of TIP-OA volunteers**<br><br>The following criteria have been selected to categorize volunteers at intake according to their past volunteering experience. This method of categorizing volunteers is similar to how we stratify clients (Appendix B) and allows for a quick and simple matching of compatible volunteers and clients.

All volunteers must be 18 years old and older<br>**Additional assets, but not required, would be prior experience with seniors, good overall level of maturity (subjectively measured) and/or lived experience	
**Code Green Volunteers:**	1. Approved background check<br>2. Some volunteer experience<br>3. New volunteers with enthusiasm to support the program
**Code Orange Volunteers:**	1. Approved background check<br>2. A minimum of **one or more** of the following criteria:<br>a. Relevant certification or degree (bachelor's in psychology, etc)<br>b. 2 or more years of experience helping people in volunteering or professional capacity
**Code Red Volunteers:**	1. Approved background check<br>2. A minimum of **one or more** of the following criteria:<br>a. 2 or more years of experience helping individuals with mental health needs<br>b. 5 or more years of experience helping others in volunteering or professional capacity<br>c. Clinician, clinician trainee, or mental health clinical research background<br>i. Med school students, retired social worker, experienced research assistant in mental health

---
