# Supplementary material for: Connecting During COVID-19: A Protocol of a Volunteer-Based Telehealth Program for Supporting Older Adults' Health
Source: Front Psychiatry. 2020 Dec 2;11:598356. doi: 10.3389/fpsyt.2020.598356 (PMC7738321; doi:10.3389/fpsyt.2020.598356)
Supplement: Supplementary file 2 [file Data_Sheet_2.PDF]

Appendix B. Client categorization according to baseline risk assessment

The following criteria have been laid out to categorize clients at baseline according to case complexity. The stratification of clients is crucial when it comes time to match them with volunteers, as the colour codes offer a quick and simple manner to match clients with volunteers who possess appropriate skills and experience. Clients are categorized as follows: Mild/Green (any client meeting mostly mild and/or "not present" criteria); Moderate/Orange (any client meeting at least two moderate/code orange criteria); and Severe/Red (any client meeting at least one severe/code red criteria).

**Symptom Categories**	**Mild** (Code Green)	**Moderate** (Code Orange)	**Severe** (Code Red)
Confusion	– Alert, grossly oriented<br>– Client presents minor lapses in memory - may forget the screener's name, or ask the same question twice<br>– Overall, the client appears mostly "with it" and only rarely repeats himself/herself	– Client is alert, but may not know the exact date<br>– Tends to repeat himself/herself excessively	– Client is quite confused, does not know who the referring clinician is despite multiple promptings (JGH, Geri Psych clinic, Dr X)<br>– The screener needs to ask the same questions multiple times (going beyond language barrier or hearing impairment issues)<br>– Unclear reliability, at times may appear incoherent
Mood (depression, anxiety)	– Client experiences periods of depression of anxiety, but these are only temporary, not overwhelming<br>– Remains able to manage well daily<br>– Enjoys pleasurable activities, able to	– Client endorses prolonged distress<br>– This level of distress starts to have an impact on the client's daily life<br>– Distress is becoming difficult to control<br>– It can be accompanied by an increase in physical/somatic	– Severely depressed mood and constant, high levels of anxiety<br>– May be unable to get out of bed, unable to eat, unable to complete ADL's<br>– Unable to manage alone

	self-soothe and distract	symptoms i.e. headache, fatigue, pain<br>– Also be on the lookout for increased help-seeking, or on the contrary, self-isolation (especially due to apathy or anxiety)	
Suicidality<br>*Any mention of suicidality across all levels will be provided with crisis hotlines to contact	– Passive thoughts of dying/death<br>– No intention to act.<br>Ex:<br>– “Some days, I wish I wouldn’t wake up in the morning.”<br>– “Sometimes I wish I would die in my sleep so I won’t have to deal with everything anymore”<br>– “You know, I envy the people who died already; they have it easier than me.”	– Active thoughts of suicide<br>– No intention to act<br>Ex:<br>– “Almost every day I wish I could die, but I won’t because of [my children, my religion, my pets]”<br>– “There doesn’t seem to be much reason for me to live anymore, but I can’t bring myself to the point of contemplating suicide”<br>– Contact the referring physician to gather more information<br>– May require higher intensity of virtual follow-up	– Active thoughts of suicide<br>– Action plan for potential suicide attempt<br>Ex:<br>– “I can't live like this anymore but if I take all my pills it will end this suffering”<br>– “I’m pretty sure that if I jumped off my balcony I would die. I think about it more and more.”<br>– Contact the referring physician/clinician on-call for guidance
Psychotic thought content (delusions, hallucinations, paranoid ideations)	– Psychotic thought content may be noted<br>– Inquire if this is impacting their daily functioning/is troublesome	– Psychotic thought content is imposing functional limitations or heightened levels of distress<br>Ex:	– Psychotic thought content is severely impacting function and elevating distress.<br>Ex:

*If psychotic thought content is present please inquire about:<br><br>-Impact on their overall well-being<br><br>-Level of conviction<br><br>-Level of preoccupation (how easily are they redirected)	throughout their day (some psychotic thought content may not be detrimental to clients' overall well-being)<br><br>Ex:<br><br>– "Most people online are trying to scam me."<br>– "I think my floormates are laughing behind my back all the time."<br>– "I think the nurses are switching people's pills."<br><br>– Able to manage with redirection or reassurance and support	– "I can't sleep at night because my neighbour is trying to break in."<br>– "I keep the water running while I'm on the phone so the microphones can't hear me talk."<br>– "I've been seeing signs around my house that I think are being left by the government."<br>– Able to manage with medication, medical support, guidance or reassurance<br><br>– May require higher intensity of virtual follow-up	– "My entire day is spent hiding from people trying to harm me in my apartment."<br>– "I've started to sleep with a knife under my bed, just in case my neighbour breaks in to poison me."<br>– "I don't turn on the lights or use the kitchen sink so the government can't track me."<br><br>– Unable to manage alone or be redirected in conversation.<br>– Contact referring physician/ clinician on-call for guidance
Other concerns e.g. substance use, communication difficulties.	*Clinician uses judgment to assess severity of other concerns based on the level of distress and/or the level of impairment they cause		
