# Supplementary material for: Connecting During COVID-19: A Protocol of a Volunteer-Based Telehealth Program for Supporting Older Adults' Health
Source: Front Psychiatry. 2020 Dec 2;11:598356. doi: 10.3389/fpsyt.2020.598356 (PMC7738321; doi:10.3389/fpsyt.2020.598356)
Supplement: Supplementary file 3 [file Data_Sheet_3.PDF]

Appendix C. Semi-structured interview guide for clients

This interview guide is used for the clients' 8-week qualitative assessment.

General information:

A. Age
B. Neighbourhood of residence: (e.g. NDG, Petite-Patrie, Plateau, Cote-des-Neiges)
C. Highest level of education: (elementary school graduate, high school grad, university – bachelor's degree, university – master's or PhD degree)
D. Marital Status: (single, married, common-law)
E. Languages spoken/comfortable conversing in:
F. Preferred language(s):
G. Do you identify as a visible minority? (Y/N)
H. What ethnic background do you identify with? (Caucasian, African, Caribbean, Hispanic, East Asian, South Asian, Southeast Asian, Indigenous, Other, Mixed)
I. Do you live with anyone currently: (alone, other family member, spouse, child, friend)
J. Do you live in a group home, supervised apartment, long-term care facility, or nursing home: (group home, supervised apartment, long-term care facility, nursing home, other – please specify)
K. Baseline Risk Level (red/yellow/green):

1. **How did you come to know about this program?**
Prompts:
a. Was the way to access information about the program convenient for you (e.g., referred by your clinician, poster, social media, friends, family, etc.)?
b. Do you think you could/should have known about it earlier?

2. **Can you please provide some information about your situation at the time when you received the phone call?**
Prompts:
a. Were you isolated and living alone?
b. Were you worried and stressed because of the Covid19 situation?
c. Did you have access to services, if yes which ones and how?
d. Did you have access to a caregiver living in the same city/neighborhood? Did you find it easy to access them or not?
e. Did you have medical or other conditions, would like to share?

3. **How was your contact experience with the telehealth program?**
Prompts:
a. Who contacted you? Did you understand correctly what was offered?
b. Were your questions answered?
c. Do you remember how many people associated with the program called you?
d. Did you like your volunteer-partner? Were you able to share your concerns with her/him?
e. Was s/he able to help you with your issues? What kind of help did you need?

4. How did the program help your overall situation?

Prompts:

a. Did you feel relaxed after the phone calls?

b. Did you feel connected to the world through this program?

c. Did this program offer you enough information? If not, what was missing?

d. Did you find the volunteer-partner dependable and accessible?

e. Do you think the number of calls per week was enough to meet your needs?

f. Did you request the service in a specific (ethnic) language? If so did you get it and was it helpful?

5. Do you have suggestions/concerns for the program team?

Prompts:

a. Do you think the team provided enough clarity and information to satisfy your needs?

b. Would you like this program to continue even after the Covid19 is over?

c. Would you be interested in this kind of program, but offering other services in the future?

d. Would you be interested in online programs for health improvement in the future by our team?

e. Any specific thing that you liked/disliked about this program?

f. How can we improve this program?

Thank you for your time.
